# A Combination of Aqueous Extraction and Polymeric Membranes as a Sustainable Process for the Recovery of Polyphenols from Olive Mill Solid Wastes

**DOI:** 10.3390/polym11111868

**Published:** 2019-11-12

**Authors:** Carmela Conidi, Agata Egea-Corbacho, Alfredo Cassano

**Affiliations:** 1 Institute on Membrane Technology, ITM-CNR, University of Calabria, via P. Bucci, 17/C, I-87030 Rende, Cosenza, Italy; c.conidi@itm.cnr.it; 2 Department of Environmental Technologies, Faculty of Marine and Environmental Sciences, University of Cadiz, 11510 Puerto Real, Cádiz, Spain; agata.egea@uca.es

**Keywords:** polyphenols, olive mill solid wastes, polymeric membranes, ultrafiltration, nanofiltration

## Abstract

Polyamide commercial membranes in flat-sheet configuration and with molecular weight cut-off (MWCO) in the range of ultrafiltration (UF) to nanofiltration (NF) were tested for the recovery of phenolic compounds from clarified olive mill solid waste (OMSW) aqueous extracts. The performance of selected membranes was evaluated in terms of productivity (permeate flux) and selectivity towards biologically active compounds (such as phenolic compounds, flavanols, and hydroxycinnamic acids derivatives) and total antioxidant activity (TAA) as a function of transmembrane pressure (TMP). NF membranes produced higher permeate fluxes and a lower fouling index in comparison with UF membranes. Retention of bioactive compounds was also significantly higher for NF membranes than for UF membranes. In particular, membranes with MWCO in the range 150–500 Da showed rejection towards flavanols and hydroxycinnamic acid derivatives of about 100%. On the other hand, the rejection towards TAA and total polyphenols was of about 90% and 72%, respectively. Therefore, NF retentate fractions appear of practical interest for the production of food additives and food supplements due to their high antioxidant activity.

## 1. Introduction

Olive processing and olive oil production are well established activities in different Mediterranean countries, including Morocco, Spain, Italy, Greece, and Turkey, accounting for about 76% of the world’s olive oil production [1]. The extraction of olive oil is accomplished by the production of large amounts of dark liquid effluents called olive mill wastewaters (OMWs) consisting of solid and liquid matter with a slightly acidic pH. The latter is composed of polyphenols, carbohydrates, fatty acids, and water. On average 0.5–0.8 m^3^ of OMWs are produced for 1 ton of treated olives. The production of these wastes, especially in the 3–4 months of intensive olive oil production, and the variability of the waste composition represent the main challenge of the olive oil industry [2].

These secondary products have an impact on soil and air quality as well as on aquatic ecosystems due to the deposition of toxic effluents directly into the receiving bodies [3,4]. Therefore, complex management and disposal systems are required in order to minimize the environmental impact and to enable a sustainable use of resources.

A combination of different physico-chemical and biological processes, including solar distillation, centrifugation, flocculation, adsorption, composting, and anaerobic/aerobic digestion, is effective for pollution control, resulting in considerable organic load and toxicity abatement [5]. However, these techniques are not used due to economic and technical reasons (i.e., high amount of sludge produced). In addition, the small scale and dispersed nature of olive mills, as well as the seasonality of the process, make it difficult to find affordable solutions to meet the quality required by environmental standards [6]. Therefore, there is a huge need to develop more efficient and economically viable technologies. On the other hand, it is well known that OMWs, if properly managed, appear to be an inexpensive source of biologically active compounds, mainly represented by phenolic compounds [7,8]. The most important biophenols in olive mill by-products include benzoic acid and hydroxycinnamic acid derivatives and, in larger amounts, tyrosol, hydroxytyrosol, and oleuropein. These compounds exhibit potential antioxidant, antimicrobial, anticarcinogenic, and skin and bone regenerative properties [9,10]. Furthermore, recent research suggests that olive phenolics could play a significant role in the prevention and treatment of different lifestyle-related diseases, including neurodegeneration [11]. Such bioactivity drives the use of these by-products for new applications, such as food additives, natural food antioxidants as alternative to synthetic ones [12], or for cosmetics [4]. Hence, the recovery of polyphenols from olive mill by-products not only improves the economic status of olive oil producers, but it also makes them less toxic and easier to treat, promoting the overall sustainability of their management. Thus, it is of great interest to evaluate the possibility of recovering extracts enriched in phenolic compounds, from a low-cost and widely available by-product, especially in the Mediterranean area [2].

The “Five-Stages Universal Recovery Processing” approach has been reported in the literature for recovering valuable compounds from food by-products including: (i) macroscopic pre-treatment, (ii) separation of macromolecules from micro-molecules, (iii) extraction, (iv) purification, and (v) product formation [13]. To accomplish these steps, different conventional technologies are available. Recently, green or clean techniques, based on reduced use of energy, short extraction time, decreased solvent consumption, overall enhancement of extraction rate, enhancement of the quality extracts, improvement of aqueous extraction processes, and improved extraction of heat sensitive compounds, have been proposed and investigated to overcome typical drawbacks of conventional methodologies [14]. Membrane processes, with their intrinsic properties, meet these requirements and have been successfully used in the treatment of OMWs devoted to the reduction of the pollution load and selective recovery of polyphenols [15]. In particular, pressure-driven membrane processes, such as microfiltration (MF), ultrafiltration (UF), nanofiltration (NF), and reverse osmosis (RO), have been largely investigated in the sequential design for the selective fractionation, purification and concentration of polyphenols from OMWs and for water recovery [6,16,17]. More recently, the possibility to recover bioactive compounds from olive leaves with pressure-driven membrane processes has been also reported [18].

On the basis of our knowledge, few studies have been reported until now on the use of membrane processes for the recovery of biologically active compounds from olive mill solid wastes (OMSWs). These wastes typically contain huge amount of phenolic compounds, which interact with suspended solids. RO polymeric membranes (BW30 from Dow Water & Process Solutions, USA) were less affected by fouling when compared with NF membranes in the treatment of olive pomace aqueous extracts [19]. They also exhibited higher rejection towards total organic carbon (99.93%), salts (99.72%), and phenolic (100%) and flavonoid (100%) compounds. An integrated approach, based on the use of UF, NF, and RO for recovering polyphenols from semi-solid wastes (pomace or alperujo), has been recently investigated by Sygouni et al. [20]. In this approach, the UF step was used to remove suspended solids, fat, lipids, and high molecular organic molecules from a hydroalcoholic extract of two-phase olive mill semi-solid waste. In the NF step, most of the organics and the total phenolic content were recovered in the retentate stream. A final RO step was used for the tertiary treatment of the NF permeate, producing a final clear effluent with limited concentrations of phenolics and organics.

This work was aimed at investigating a sustainable process for the purification and recovery of polyphenols from OMSWs. It was based on an aqueous extraction step of the olive waste followed by pre-treatment of the extract by MF and a fractionation/concentration step of the MF permeate through the use of tight ultrafiltration (UF) and nanofiltration (NF) membranes. Permeate fluxes, fouling index, cleaning efficiency, and selectivity towards biologically active compounds (such as phenolic compounds, flavanols, and hydroxycinnamic acids derivatives) and total antioxidant activity (TAA) were analyzed and discussed for each investigated membrane.

## 2. Materials and Methods

### 2.1. Materials

Olive mill wastewaters, obtained through a three-phase process, were supplied by Olearia San Giorgio (San Giorgio Morgeto, RC, Italy). Olive mill solid wastes were obtained after treatment of olive mill wastewaters with sulfuric acid until pH 2.5 in order to achieve the coagulation and precipitation of undesired solids according to Stokes’ law.

Gallic acid (MW 170.12 g/mol), Folin–Ciocalteu phenol reagent, potassium persulfate (MW 270.3 g/mol), 6-hydroxy-2,5,7,8-tetramethylchroman-2-carboxylic acid (Trolox) (MW 250.29 g/mol), 2,20-azinobis(3-ethylbenzothiazoline-6-sulfonic acid) diammonium salt (ABTS) (MW 548.68 g/mol), caffeic acid (MW 180.16 g/mol), and hydrochloric acid (37%) were from Sigma Aldrich (Milan, Italy). Sodium carbonate anhydrous (MW 105.99 g/mol), sodium chloride, sodium hydrogen, and dihydrogen phosphate were supplied by Carlo Erba (Milan, Italy).

### 2.2. Extraction of Phenolic Compounds from Olive Mill Solid Wastes

The optimization of aqueous extraction of polyphenols from OMSWs was performed at lab scale in terms of liquid-to-solid ratio (L/S) (from 5 to 20 mL/g) and extraction temperature (from 30 to 70 °C), at a selected extraction time of 60 min. The extraction was performed in water as it is safer, environment-friendly, and less expensive than alcohols or other solvents, so able to guarantee a better food product quality [21].

Aqueous extracts, produced under operating conditions optimized at lab scale, were stored in the freezer (−20 °C) until usage.

### 2.3. MF Pre-treatment of OMSW Extracts: Equipment and Procedures

The aqueous extract was microfiltered using a laboratory pilot unit equipped with a polypropylene membrane module in tubular configuration (MD 020 TP 2N, from Mycrodin Nadir, Wuppertal, Germany) with a nominal pore diameter of 0.2 μm and a membrane surface area of 0.036 m^2^. The MF system was operated at a transmembrane pressure (TMP) of 0.32 bar, and a feed flowrate (*Q_f_*) of 400 L·h^−1^ and a temperature (T) of 27 ± 2 °C according to the batch concentration mode (recycling the retentate stream and collecting separately the permeate). Experiments were performed in triplicate and permeate fluxes were expressed as the average value ± SD. Membrane cleaning was performed by using an enzymatic detergent (Ultrasil 50, Henkel Chemicals Ltd., Dusseldorf, Germany) at a concentration of 1% (w/w), followed by cleaning with a 0.1% (w/w) NaOH solution. Cleaning solutions were re-circulated in the MF plant for 60 min at a temperature of 40 °C. At the end of each cleaning procedure the membrane module was rinsed with distilled water for 20 min and the water permeability was measured.

### 2.4. Treatment of Clarified OMSW Extracts with UF and NF Membranes: Equipment and Procedures

Clarified extracts were processed by UF and NF polymeric membranes. Experiments were performed by using a laboratory bench plant supplied by Three-Es Srl (Milano, Italy). The plant consisted of a feed tank, a positive displacement pump (Cut pump, pressure range 7–140 bar, maximum operational flow 16 L/min), a stainless steel cross-flow cell having a filtration area of 0.0032 m^2^, a digital flow meter, two pressure gauges (0–60 bar) for measuring of the inlet and outlet pressures, a pressure control valve, and a cooling coil fed with tap water used to maintain the feed temperature constant. An inverter connected to the pump was used to control the pumping velocity. The permeate flux (J) was determined by measuring the collected permeate volume at a given time through the membrane surface area.

UF and NF experiments were performed according to the total recycle configuration in which both permeate and retentate streams were continuously recycled to the feed tank in order to study the effect of transmembrane pressure (TMP) on the permeate flux and selectivity towards the compounds of interest. TMP was modified in the range 5–25 bar at fixed conditions of feed flow rate (*Q_f_* = 498 L/h) and temperature (26 ± 1 °C).

For each membrane, the permeate flux was measured by increasing the TMP value from the lowest to the highest one. Once the stable flux value was reached at a given TMP, the pressure was increased to the next value. Samples of feed, retentate, and permeate were collected for each investigated TMP and stored at −20 °C until analyses.

Approximately 1.5 L of clarified extracts were used for each experiment.

Five thin-film composite flat-sheet membranes with different average molecular weight cut-offs (MWCOs) (from 150–300 to 3500 Da) were used. Their typical characteristics according to the manufacturers’ data sheet are reported in Table 1.

A burette cylinder was used to average the volume variation at the output of the permeate stream in a fixed time interval. The permeate flux (*J_p_*), expressed as L/m^2^h, was determined by measuring the collected permeate volume (*V_p_*) at a given time (*t*) through the membrane surface area (*A*) according to the following equation:

(1) $$J_{p}=\frac{V_{p}}{A\cdot t}.$$

**Figure 1:**
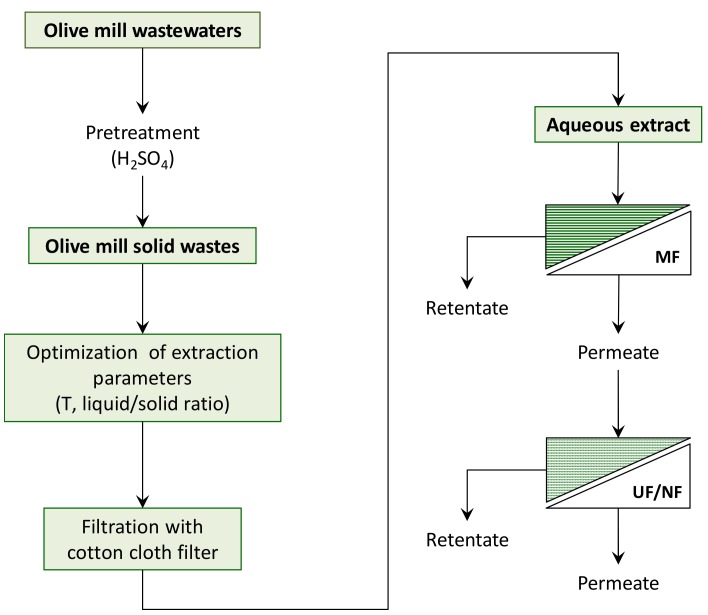
Schematic representation of the investigated process of extraction and membrane-based treatment of olive mill solid waste (OMSW).

A schematic representation of the investigated process of extraction and membrane-based treatment of OMSW is illustrated in Figure 1.

### 2.5. Measurement of Hydraulic Permeability and Membrane Cleaning

Before each experiment, the water permeability (*L_p0_*) of each membrane was determined by the slope of the straight line obtained by plotting the water flux values, measured in fixed conditions of temperature (25 °C), as a function of the TMP, according to the Darcy’s law [24]:

(2) $$L_{p}=\frac{J_{w}}{TMP},$$

where *J_w_* is the pure water flux (L/m^2^h).

The fouling index (*I_f_*), expressed as a percentage drop in the water permeability, was estimated by measuring the water permeability before and after the treatment of OMSW extracts, according to the following equation [25]:

(3) $$I_{f}=\left(1-\frac{L_{p1}}{L_{p0}}\right)\cdot 100,$$

where *L_p0_* and *L_p1_* are the water permeabilities measured before and after the treatment of OMSW extracts, respectively.

After the treatment with OMSW extracts, the selected membranes were cleaned in two steps. The first cleaning step was performed by recirculating tap water for 15 min through the membranes in order to remove the reversible polarized layer. In the second step, the membrane module was submitted to a cleaning with an enzymatic solution (1% w/w Ultrasil 50) at 40 °C for 60 min. At the end of each cleaning procedure, membranes were rinsed with tap water for 15 min and the hydraulic permeability was measured once again (*L_p2_*).

Flux recovery (*FR*, %) was calculated according to the following equation:

(4) $$FR=\left(\frac{L_{p2}}{L_{p0}}\right)\cdot 100.$$

### 2.6. Analytical Methods

Feed, permeate, and retentate samples coming from each membrane treatment were analyzed for suspended solids, total antioxidant activity, and phenolic compounds.

The rejection (*R*) of selected membranes toward specific compounds was determined as:

(5) $$R=\left(1-\frac{C_{p}}{C_{f}}\right)\cdot 100,$$

where *C_p_* and *C_f_* are the concentration of a specific component in the permeate and feed streams, respectively.

The suspended solid content was determined in relation to the total extracts (%, w/w) by centrifuging, at 2000 rpm for 20 min, 45 mL of a pre-weighted sample; the weight of settled solids was determined after removing the supernatant.

Total polyphenols were measured by the Folin–Ciocalteu method [26]. The method is based on the reduction of tungstate and/or molybdate in the Folin–Ciocalteu reagent by phenols in alkaline medium resulting in a blue colored product (λmax 756 nm). The estimation of total phenols was carried out in triplicate and results were expressed as mg/L gallic acid (mg GAE/L).

The determination of different polyphenol classes was performed according to the method reported by Obied et al. [27]. One mL of diluted ethanolic extract (1:10 in water) was mixed with 1 mL of HCl-ethanol solution (0.1 mL HCl/100 mL in 95 mL ethanol/100 mL) into a 10 mL volumetric flask and the volume was made up to 10 mL with 2 mL HCl/100 mL. After mixing, the absorbance was measured at 320 and 360 nm to determine hydroxycinnamic acid derivatives and flavanols, respectively. Results were expressed as mg/L of caffeic acid (λ, 320 nm) and quercetin (λ, 360 nm), respectively.

TAA was determined by an improved version of the ABTS assay in which the radical cation is generated by reaction with potassium persulfate before the addition of the antioxidant (decolorization assay) [28]. This method gives a measure of the antioxidant activity of pure substances and of mixtures by monitoring the reduction of the radical cation as the percentage inhibition of absorbance at 734 nm. Spectrophotometric measurements were performed by using a UV–Visible recording spectrophotometer (UV-160 A, Shimadzu Scientific Instruments, Inc., Japan) at 30 °C. Results were reported as mmol Trolox equivalent.

Analytical determinations were performed in triplicate. Results were expressed as the mean ± SD.

## 3. Results and Discussion

### 3.1. Aqueous Extraction of OMSWs

The effectiveness of the extraction process of polyphenols is largely regulated by different experimental parameters including the extraction temperature. Indeed, several studies have indicated that temperature is one of the most important parameters affecting the extraction efficiency of polyphenols from plant materials [29,30]. Basically, an increase of temperature leads to an increase of the diffusion rate and solubility of the extracted substances. On the other hand, it should also be considered that bioactive compounds, such as polyphenols, are damaged at high temperatures [31]. Therefore, to investigate the impact of temperature on the yield and stability of the polyphenols, a range of temperatures from 20 to 70 °C, was applied.

**Figure 2:**
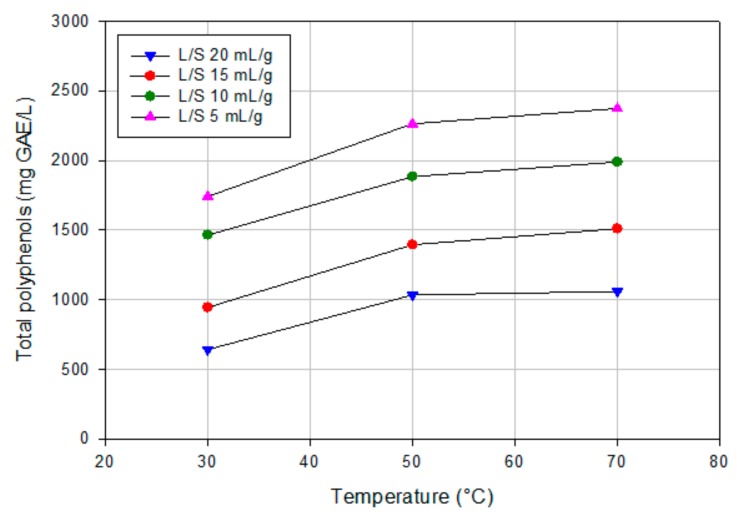
Influence of temperature and liquid-to-solid ratio (L/S) on polyphenols extraction from OMSWs.

The profiles of total polyphenols extracted from OMSWs at different temperatures are reported in Figure 2. As expected, the total polyphenol content increased gradually by increasing the temperature in the range of investigated values (30–70 °C). Therefore, the optimal extraction temperature of polyphenols was selected as 70 °C. These results are in agreement with those reported by Alu’datt et al. [32] who analyzed the extraction conditions of polyphenols from olive cake. In particular, the authors studied the effect of extraction temperature (from 25 to 70 °C) on total polyphenols, obtaining the highest extraction value at 70 °C. Optimal extraction temperature was chosen to be 80 °C in the extraction of catechins from green tea using hot water [33]. As previously discussed, high temperatures decreased solvent viscosity and improved diffusion and, consequently, mass transfer of solutes, including polyphenols.

The L/S ratio is considered another important factor that affects the extraction of biocompounds from vegetable matrices [34]. According to Figure 2, an increase in polyphenol concentration was observed by decreasing the L/S ratio from 20 to 5 mL/g for all investigated temperatures; this means that the polyphenols extracted from OMSWs are diluted when a higher solvent volume is used. An increase of total polyphenols content by decreasing the solid to liquid ratio (S/L, from 1:10 to 1:5) was also observed by Stamatopoulos et al. [35] who investigated the effect of S/L ratio on the recovery of phenolic compounds from olive leaves. Although the extraction yield increased by increasing the L/S ratio (at 70 °C the concentration of polyphenols expressed as mg GAE/g OMWS was 11.8 for a L/S ratio of 5 mL/g and 21.2 for a L/S ratio of 20 mL/g, respectively), an L/S ratio of 5 mL/g was selected in order to reduce the volume of water needed for the extraction. Indeed, the volume of required solvent determines the size of the extraction unit; a lower volume of solvent is preferable because not only less water is needed for the extraction but also less energy is required for heating it up [36].

### 3.2. Microfiltration of OMSW Aqueous Extract

**Figure 3:**
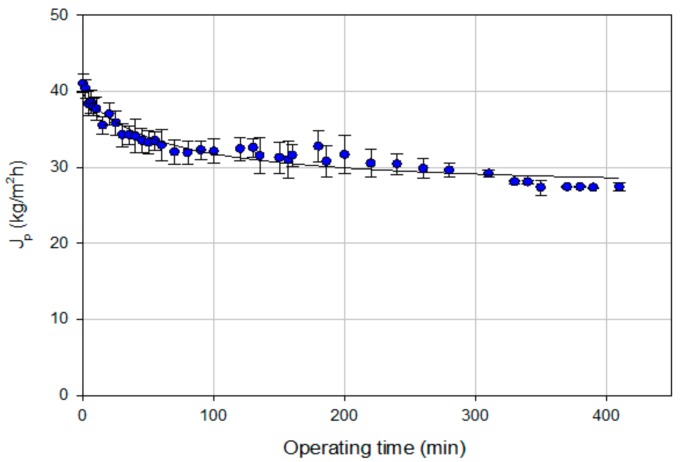
Microfiltration of OMSW aqueous extracts. Time course of permeate flux (operating conditions: T, 27 ± 2 °C; TMP, 0.32 bar; *Q_f_*, 400 L/h).

The aqueous extract obtained in the optimized extraction conditions (70 °C, L/S 5 mL/g, and 60 min) was clarified by MF. Figure 3 shows the time course of permeate flux related to the clarification of the aqueous extract by MF in the selected operating conditions up to a recovery factor of 80%. The results showed a decrease of permeate flux in the first minutes, followed by a smooth gradual decline until the end of the process. In particular, the initial permeate flux of about 42 kg/m^2^h decreased with the operating time until it reached a steady-state higher than 28 kg/m^2^h. According to several authors, the decline of permeate flux can be attributed to concentration polarization and fouling phenomenon as well as to the increase of the solute concentration in the retentate. Pore blocking phenomena are generally involved in the first steps of the filtration process while the gradual slow flux decline is caused by the accumulation of foulant molecules on the membrane surface, with a formation and consolidation of a cake layer or a gel layer on the membrane surface [37,38].

In Table 2 the chemical composition of permeate and retentate streams obtained in the MF treatment of the aqueous extract is reported. It is well known that olive mill wastes are considered to be an interesting source of phenolic compounds, the most important being phenolic alcohols, phenolic acids, lignin, and flavonoids. A high correlation between these individual and combined phenolic compounds and the antioxidant activity, was found by Suárez et al. [39]. The aqueous extract was characterized by a total antioxidant activity (TAA) of 11.2 of Trolox and a total polyphenol content of 1812 mg/L of gallic acid. These values were higher than those reported in olive mill wastewaters [40]. Similarly, the content of flavanols and hydroxycinnamic acid derivates (190.4 mg/L and 180.2 mg/L, respectively) was greater than the amount reported by Galanakis et al. [41] in olive mill wastewaters. The MF membrane allowed for the preservation of all the analyzed bioactive compounds and TAA in the permeate side due to the low rejection of the membrane towards these components (in the range 1.8%–7.7%). On the other hand, suspended solids were completely removed with a production of a clear extract.

### 3.3. Treatment of Aqueous Extract with UF and NF Membranes: Effect of TMP on Permeate Flux

**Figure 4:**
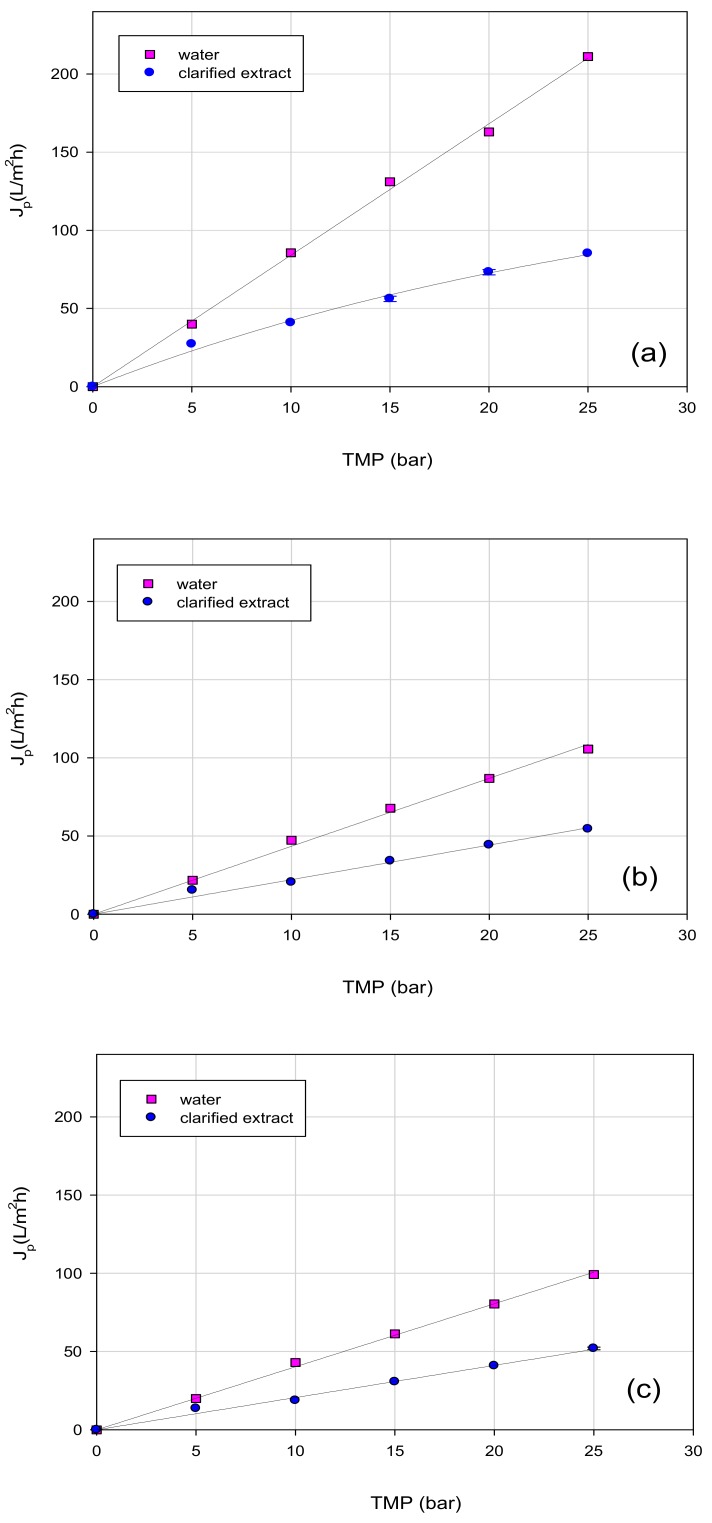
Effect of TMP on water flux and steady-state permeate flux of OMSW extracts for UF membranes: (**a**) GK membrane (MWCO, 3500 Da); (**b**) GH membrane (MWCO, 2500 Da); (**c**) GE membrane (MWCO, 1000 Da).

**Figure 5:**
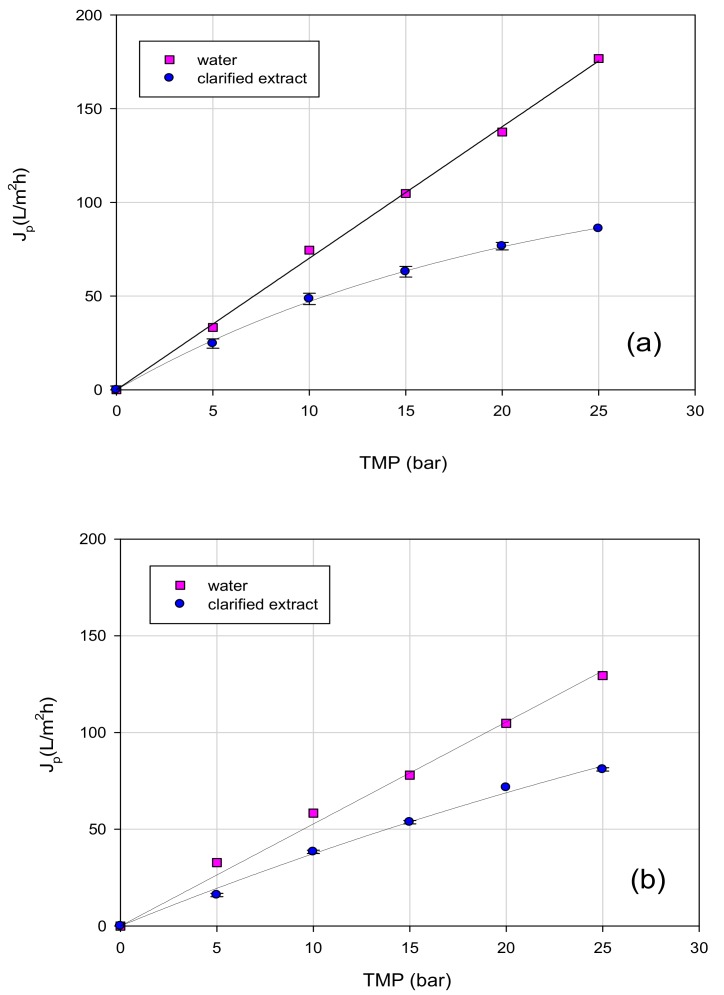
Effect of TMP on water flux and steady-state permeate flux of OMSW extracts for NF membranes: (**a**) NFA-12 membrane (MWCO, 500 Da); (**b**) DK membrane (MWCO, 150–300 Da).

The dependence of permeate flux as a function of the TMP for both clarified extract and water with UF and NF membranes is reported in Figure 4 and Figure 5, respectively. It can be seen that pure water fluxes increased linearly by increasing the TMP for each tested membrane following Darcy’s law. However, during the treatment of clarified extract, two different trends of flux as a function of the TMP were identified. GH and GE membranes (with MWCO of 2500 and 1000 Da, respectively) showed a linear relationship between flux and applied pressure, despite that the permeate flux values were lower than those measured with water. For both membranes, the difference in the permeate fluxes between the clarified extract and water was greater at higher operating pressures.

For GK and NF membranes, the permeate flux increased linearly with TMP only at low pressures (up to 10 bar); at a higher TMP, the permeate flux showed a deviation from a linear flux-pressure behavior and it became independent of pressure. However, in the range of investigated values (5–25 bar) a limiting flux was not achieved at all. Similar results were obtained by Díaz-Reinoso et al. [42] in the treatment of aqueous extracts from pressed distilled grape pomace with UF and NF membranes: operating with the GE membrane, a linear interrelationship between TMP and permeate flux was observed, while for a polyamide NF membrane with a MWCO of 350 Da (Nanomax 50, Millipore) a higher TMP led to independent permeate flux values for further increases in pressure. Non-linear increases of permeate fluxes with TMP were also observed by Xu and Wang [43], in the concentration of flavones by NF of *Ginkgo biloba*, and by Giacobbo et al. [44], in the treatment of a microfiltered extract of red wine lees with composite fluoro polymer UF membranes (ETNA 01PP and ETNA 10PP, from Alfa Laval) with an MWCO of 1 and 10 kDa (ETNA 01PP and ETNA 10PP, respectively, from Alfa Laval).

As observed in Figure 4 and Figure 5, the permeate flux did not vary proportionally with the MWCO of the selected membranes, suggesting that other parameters, including hydrophilicity/hydrophobicity, are crucial factors in membrane performance. Hydrophilic membranes hold water better, thus facilitating flux through pores [45]. The highest fluxes with the clarified extract were achieved with the NF 12A membrane (with MWCO of 500 Da), which presented the lowest contact angle value, whereas the lowest permeate fluxes were observed for GH and GE membranes (with MWCO of 2500 and 1000 Da, respectively). For UF membranes, small particles with the same size range of membrane pore size can clog membrane pores, increasing the resistance to the flow. On the other hand, these particles would remain out of the pores of NF membranes resulting in a comparatively lower flow resistance [42].

These results were in agreement with those related to the fouling index and water flux recovery (Table 3). In particular, the fouling index decreased by decreasing the MWCO on the investigated membranes: NF membranes showed the lowest fouling index (12.5% and 29.6% for DK and NFA 12 A membranes, respectively) with the highest water flux recovery (higher than 97%) after the chemical cleaning. Fouling increases with membrane pore size, indicating that pore blocking could be a major contributor to membrane fouling [46].

### 3.4. Treatment of Aqueous Extract with UF and NF Membranes: Effect of TMP on Solute Rejection

**Figure 6:**
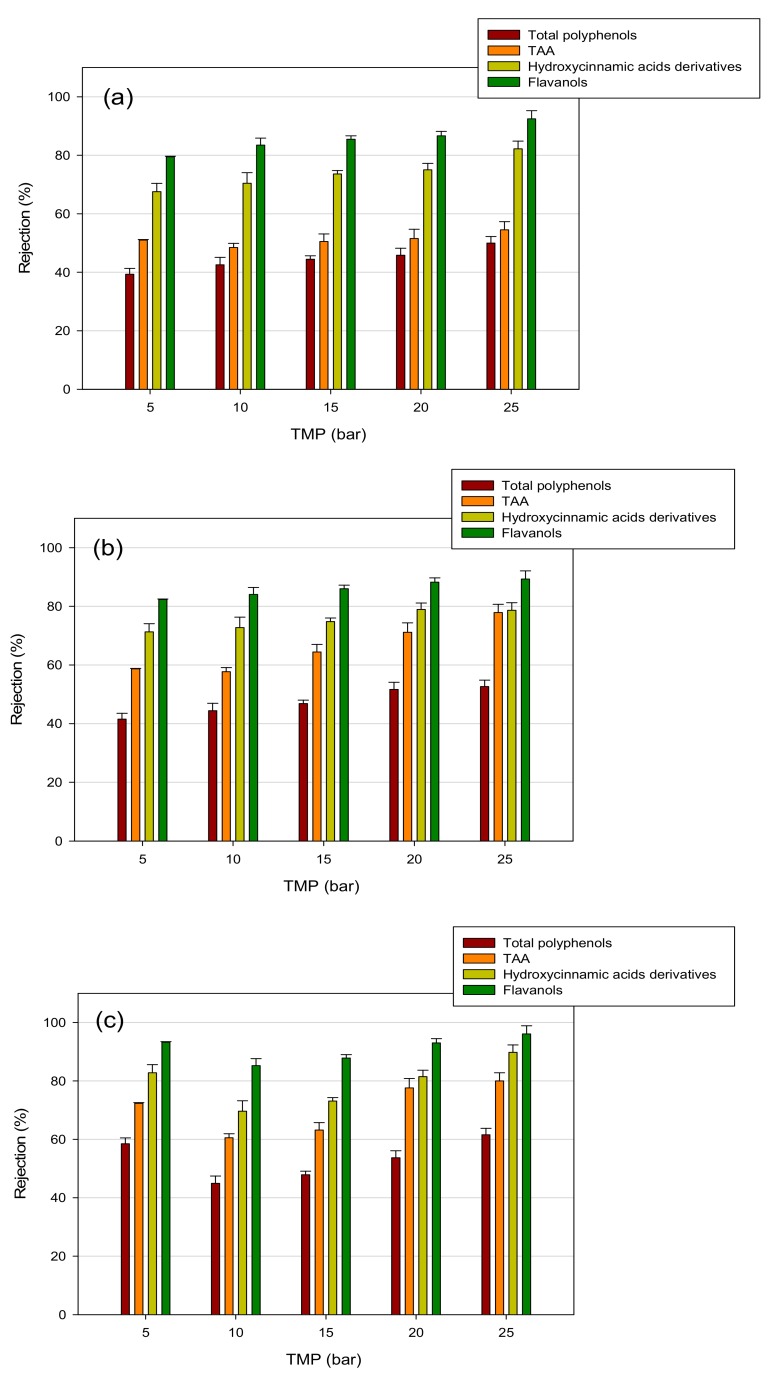
Rejection of UF membranes towards analyzed compounds: (**a**) GK membrane (MWCO, 3500 Da); (**b**) GH membrane (MWCO, 2500 Da); (**c**) GE membrane (MWCO, 1000 Da).

**Figure 7:**
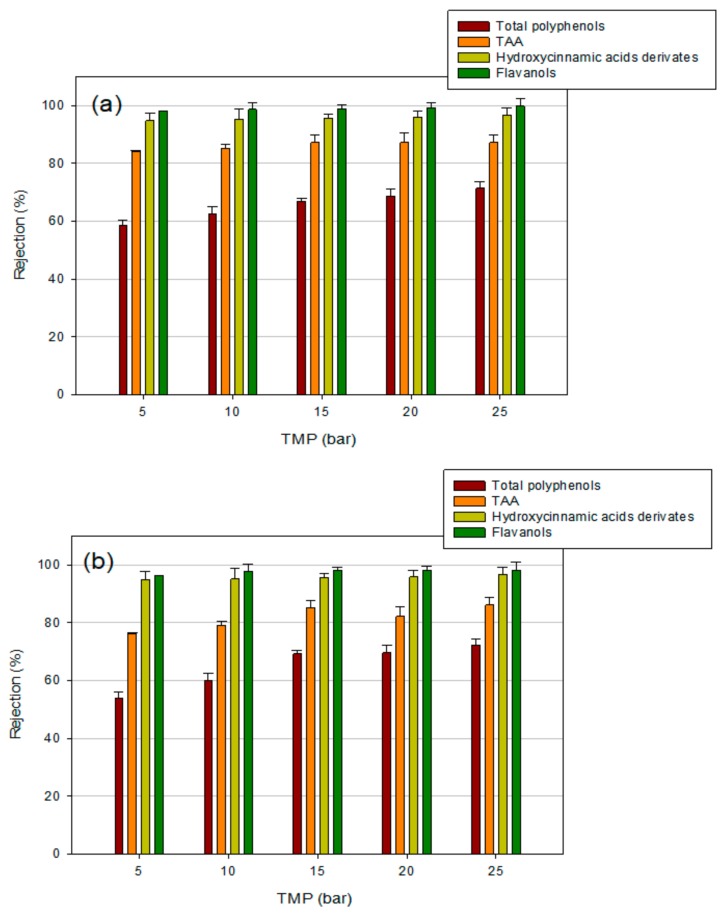
Rejection of NF membranes towards analyzed compounds: (**a**) NFA-12 membrane (MWCO, 500 Da); (**b**) DK membrane (MWCO, 150–300 Da).

In Figure 6 and Figure 7, the rejection values of selected membranes as a function of the TMP are reported. As a general trend, an increase in the rejection towards all the analyzed compounds by increasing the applied TMP was observed. In particular, UF membranes, with a higher MWCO, presented a greater increase in bioactive compounds rejection with the increase in TMP, in comparison with NF membranes. This behavior could be attributed to a more severe concentration polarization and membrane fouling when the TMP is increased, leading to the formation of a cake layer on the membrane surface and, therefore, to an increased rejection coefficient [47]. Membrane compaction inducing pore size reduction can also occur at higher operating pressures [48]. Therefore, retentates produced at higher TMP values resulted in better quality due to the enriched content of bioactive compounds. These results are consistent with those reported by other authors. Díaz-Reinoso et al. [49] reported an increase in polyphenol rejection by increasing the TMP in the treatment of white vinasse with UF and NF membranes. Similarly, Cissé et al. [50] found an increased rejection of anthocyanins with TMP in the processing of roselle extract (*Hibiscus sabdariffa* L.) through polymeric membranes with MWCO in the range 0.2–150 kDa.

**Figure 8:**
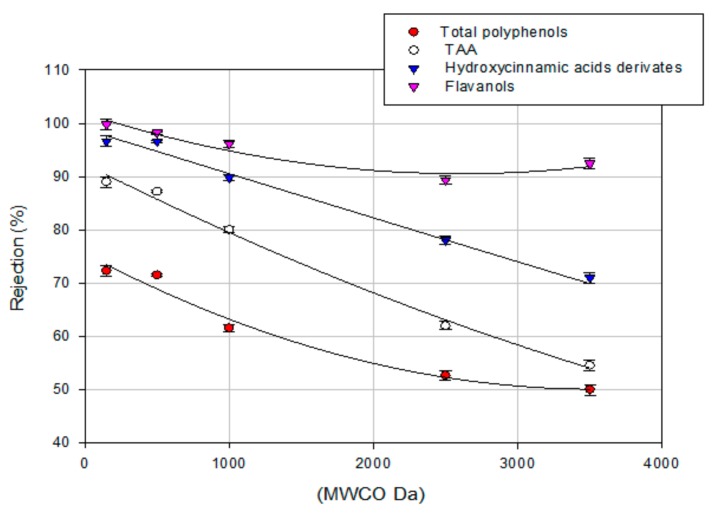
Effect of MWCO on the rejection of analyzed compounds.

Figure 8 summarizes the effect of the MWCO on the rejection towards biologically active compounds and TAA for the selected UF and NF membranes at a TMP of 25 bar. For all compounds, the rejections increased by decreasing the MWCO, allowing for the prediction of rejection values according to the membrane’s MWCO. Therefore, the physical sieving of solutes larger than the membrane MWCO gives a significant contribution to the retention mechanism of target compounds [51]. In particular, membranes with MWCO in the range 150–500 Da showed rejection towards flavanols and hydroxycinnamic acid derivatives of about 100%. On the other hand, the rejection towards TAA and total polyphenols was about 90% and 72% %, respectively. Khemakhem et al. [18] reported similar rejections values of biologically active compounds and TAA in the treatment olive leaf extract with an NF membrane of 300 Da. In the range 1000–3500 Da, typical of UF membranes, the rejections towards total polyphenols decreased from 62% to 50%; accordingly, the TAA rejection decreased from 80% to 55% in the same range. A decrease in the rejection of hydroxycinnamic acid derivatives from 90% to 71% was also observed, while the rejection of flavonoids resulted higher than 90% independent of the MWCO value.

The whole results confirmed that the retention of bioactive compounds was significantly higher for NF membranes than for UF membranes. Higher retention capacities of sugars, organic acids, and anthocyanins were also measured for NF membranes with MWCO of 150–400 Da in comparison with tight UF membranes in the treatment of roselle extract [50]. Therefore, NF membranes appear to be suitable for the concentration of aqueous extracts from OMSWs, producing concentrated fractions enriched in biologically active compounds with a high antioxidant potential. These fractions may act as antioxidant food additives and as food supplements, endowing new and relevant properties to foodstuffs and also to beverages [12,52]. Like synthetic antioxidants, and contrary to vitamin E and ascorbic acid, olive biophenols are stable at high temperatures and, at the dosage typically required (50–100 ppm), they do not interfere with taste or color of foodstuffs [11].

## 4. Conclusions

A combination of aqueous extraction and membrane-based operations was investigated on a laboratory scale for the recovery of phenolic compounds from olive mill solid wastes. The aqueous extract was preliminary clarified by MF. UF and NF polyamide membranes in flat-sheet configuration were evaluated for their performance in the processing of the clarified extract at different operating pressures.

Maximum total polyphenols yield (2350 mg/L gallic acid) was achieved in optimized conditions of the aqueous extraction (70 °C and water-to-solid-ratio (L/S) of 5 mL/g). Suspended solids were totally removed in the MF step while most parts of the biologically active compounds were recovered in the MF permeate.

NF membranes showed higher permeation fluxes and lower fouling index when compared with UF membranes. In addition, these membranes exhibited high rejection coefficients to biologically active compounds and total antioxidant activity. In particular, membranes with MWCO in the range 150–500 Da retained totally flavanols and hydroxycinnamic acid derivatives and more than 70% of total polyphenols at an optimal operating pressure of 25 bar. Therefore, NF retentate fractions could be of practical interest for the industrial production of functional ingredients due to their high antioxidant activity. Further research activities performed on a large scale and in typical concentration operative mode are needed in order to evaluate the economics of the process at industrial level.

## Figures and Tables

**Table 1:** Characteristics of ultrafiltration (UF) and nanofiltration (NF) membranes (PA-TFC, polyamide – thin film composite).

Membrane Type	GK	GH	GE	NFA-12A	DK
Manufacturer	GE Osmonics	GE Osmonics	GE Osmonics	Parker	GE Osmonics
Membrane material	PA-TFC	PA-TFC	PA-TFC	PA-TFC	PA-TFC
Configuration	flat-sheet	flat-sheet	flat-sheet	flat-sheet	flat-sheet
Nominal MWCO (Da)	3500	2500	1000	500	150-300
pH operating range	2–10	2–10	2–10	3–11	3–9
Max. operating temperature (°C)	50	50	50	63	50
Max. operating pressure (bar)	27.6	27.6	27.6	30.6	41
Membrane surface area (m^2^)	0.0035	0.0035	0.0035	0.0035	0.0035
Contact angle (°)	<61 ^a^	<61 ^a^	50 ^b^	10 ^b^	41 ^a^

^a^ Acero et al. [22]. ^b^ Cordoba et al. [23].

**Table 2:** Physico-chemical characteristics of feed, permeate, and retentate samples obtained in the treatment of OMSW aqueous extracts with the MF membrane.

Parameters	Feed	Permeate	Retentate
Total suspended solids (%)	5.4 ± 0.2	n.d.	8.6 ± 0.62
Total polyphenols (mg GAE/L)	1812.4 ± 12.6	1672.2 ± 22.6	2046.0 ± 18.6
Flavanols (mg/L quercetin)	190.4 ± 14.8	180.2 ± 11.3	205.1 ± 12.9
Hydroxycinnamic acid derivatives (mg/L caffeic acid)	180.2 ± 4.6	168.1 ± 10.4	210.2 ± 3.6
TAA (mM Trolox)	11.2 ± 1.4	11.0 ± 0.6	12.3 ± 1.2

**Table 3:** Hydraulic permeability, fouling index, and flux recovery values of UF and NF membranes.

	Membrane Type				
	GK	GH	GE	NFA-12A	DK
*Lp_0_* (L/m^2^hbar)	10.85	4.66	4.21	9.97	5.44
*Lp_1_* (L/m^2^hbar)	4.76	2.61	2.73	7.02	4.76
*L_p2_* (L/m^2^hbar)	8.19	4.34	3.52	9.97	5.27
Fouling index (%)	46.2	44.1	35.2	29.6	12.5
Flux recovery (%)	92.5	93.1	83.6	100	96.9
